# Dr. S. Lewis’ Case in Which Alcohol Was Detected in the Brain

**Published:** 1842-05-07

**Authors:** Samuel Lewis


### MEDICAL EXAMINER.

NEW SERIES.

No. 19.] PHILADELPHIA, MAY 7, 1842. [Vol. I.

### *A Case in which Alcohol was detected in the Brain—with Remarks.*

By Samuel Lewis, M. D.

To the Editors of the Medical Examiner.

Gentlemen,—During my late residence, as house surgeon, in the Royal Infirmary, Edinburgh, the following case came under my care. It may, perhaps, be deemed of sufficient interest to deserve a place in your valuable journal.

Your obt. servt.,

Saml. Lewis, M. D.

*Philadelphia, April 22d,* 1842.

Walter Smith, ætatis 45, (vagrant.) This man was admitted into the Royal Infirmary on the evening of the 8th of June, 1840. He was brought in by the police, who stated that he had been found, about half an hour previously, at the foot of a flight of steps in one of the wynds, in the neighbourhood of the hospital. No further history of the case could then be obtained. On admission he was in a perfect state of coma, with laborious breathing, cold extremities, contracted pupils, and the pulse, at the wrist, barely perceptible. The countenance was pale and turgid ; a good deal of frothy fluid was discharged from the mouth ; and the *breath had a strong alcoholic odour.* No injury could be detected on any part of the body. An emetic was given immediately, and hot applications applied to the extremities. The emetic produced no effect ; and before the stomach-pump could be introduced the patient died,—having lived about twenty minutes after admission.

On questioning his wife next day, I learned that he had left home on the previous morning quite well, on one of his usual begging expeditions ; that he had, for a long time back, been in the daily habit of getting "free," i. e. drunk ; and that lately, on one of these occasions, he had been so bad as to require the use of the stomach-pump.

*Sectio Cadaveris thirty-six hours after death.*

*Head.*—On removing the calvarium, the vessels on the surface of the brain were observed to be more than usually filled with dark blood; and there was an effusion of serum under the arachnoid sufficient to elevate that membrane above the surface of the convolutions, at the middle and posterior parts of the brain. The cerebral substance was firmer than natural, and presented an unusual number of bloody points when cut into. About an ounce of serum was found in the ventricles.

*Chest.*—The posterior part of the *left* lung was gorged with blood ; and the bronchial tubes contained a very considerable quantity of a frothy fluid. The heart was of its normal size, but the right side, as well as the ascending and descending cavæ, were filled with dark fluid blood.

*Abdomen.*—The stomach contained eight ounces of fluid, which was thought, by some present, to smell of malt liquor. The mucous membrane, throughout its whole extent, presented a healthy appearance. The liver and other abdominal viscera were free from disease. No alcoholic odour could be detected in the brain, or in any other part of the body.

In this case the patient, when brought into the hospital, seemed labouring under the effects of excessive intoxication, and was treated accordingly ; but neither the history of the case, nor the appearances after death, were sufficient to authorize a *decided* opinion in the affirmative. The alcoholic odour detected on his admission, and the account given by his wife of his fondness for strong drink, certainly favoured such a supposition, but were far from being conclusive evidence that death was caused by poisoning from alcohol.

As it was considered of some importance to ascertain, if possible, the cause of death ; and as it had lately been shown that alcohol could be easily detected, by chemical analysis, in the brains of animals, when poisoned by alcoholic liquors,* I determined to endeavour to settle the question in this way. I accordingly, in the afternoon of the same day, subjected about eight ounces of the brain to analysis, (in the manner to be mentioned more particularly presently,) having added previously a small quantity of water. After a drachm and a half of the liquid was distilled over in a test tube, the operation was discontinued, and an adequate quantity of sub-carbonate of potassa (well dried) introduced into the tube, which was then hermetically sealed, and the contents well shaken. The mixture was allowd to settle, and after a little while a stratum of a very mobile, oily-looking fluid was distinctly observed floating on the saturated solution of potassa, separated, however, from it by some ash-coloured flocculi. In order to prove that this supernatant stratum was alcohol, it was tested next morning in the presence of Drs. Reid and Low, both by flame and camphor. It burned readily with a blue flame, and dissolved camphor rapidly, leaving little or no doubt as to the nature of the fluid.

In making this analysis, (in which I was so fortunate as to obtain the assistance of Dr. Thomas Anderson, of Leith) the directions given by Dr. Percy in his Thesis, already referred to, were strictly observed. These directions I shall now give in Dr. Percy's own words ; and I do this the rather as the essay may not be easily obtained in this country, in consequence of its being chiefly published for private circulation.

"As the success of the operation," observes Dr. Percy, "in a great measure depends upon the manipulation, it is necessary to give a detailed description of the apparatus employed, and the precautions to be observed in conducting the process. Let us suppose, then, that it is required to examine some particular tissue, or, for example, the brain of a dog, in which we suspect alcohol. The entire brain, after being carefully removed, is cut into slices, which are transferred as quickly as possible into a small mattrass ; a quantity of water, sufficient to cover the whole organ thus sliced, being next poured into the vessel, a bent glass tube is adapted to the mattrass, and distillation commenced over an Argand lamp. The distillation may also be effected in the chloride of calcium bath without the addition of water to the matter subjected to analysis. As soon as ebullition begins, the operator must attentively watch the mattrass, and so regulate the heat as to prevent any portion of the

* Vide Dr. Percy's Prize Thesis "On the presence of alcohol in the brain." *Edinburgh*, 1839.

froth that is generated from passing into the recipient. When about ℥ss is distilled over, the operation may be discontinued. The product is introduced into a small test tube, containing an adequate proportion of sub-carbonate of potassa. The tube is then hermetically sealed, and, after brisk agitation, is allowed to remain at rest. If alcohol be present in appreciable quantity, it will be found collected into a stratum at the surface of the saturated solution of potassa; from which, however, it will generally, if not always, be separated by some ash-coloured flocculi. On the contrary, if alcohol be not present in appreciable quantity, there will *generally* be no appearance of any supernatant stratum of liquid, although the flocculent matter may have accumulated at the surface as usual."

It now remains to describe the manipulation which is practised in investigating the properties of the supernatant stratum of liquid eliminated, as above, by the subcarbonate of potassa. The top of the tube being broken off, the fine extremity of a capillary pipette is carefully introduced just below the surface of the contained liquid, when, in obedience to the law of capillary attraction, a portion will rise in the pipette, which may afterwards be withdrawn; in this manner a sufficient quantity of the stratum may be readily obtained for examination. In order to prove whether it have the property of inflammability, the fine extremity of the pipette is to be placed near the edge of the flame of a lamp or candle, when the liquid, which is then to be gently propelled by blowing at the other extremity of the pipette, will, if alcohol, instantly take fire and burn with a blue flame. To make the application of the camphor test, a drop of the supernatant stratum is to be removed by means of the pipette, and deposited on a small fragment of camphor placed in the field of a simple microscope. If the liquid be alcohol, it will immediately be traversed in all directions by currents proceeding from the camphor, which will, in this case, be quickly dissolved; but the alcohol will rapidly evaporate, and leave a well defined patch of camphor, consisting of little masses mixed with plumose crystallization.

The above is, I believe, only the second case in which alcohol has been detected in the brain by chemical analysis. We have, however, many cases on record where it is *stated* to have have been found in the *fluid of the ventricles*, after death from poisoning by alcoholic liquors. A few of these may be cited. Dr. Cooke, in his treatise on nervous diseases, states, that he had been informed by Mr. Carlisle, "that a few years since a man was brought dead into the Westminster Hospital, who had just drunk a quart of gin for a wager. The evidences of death being quite conclusive, he was immediately examined, and within the lateral ventricles of the brain was found a considerable quantity of limpid fluid, distinctly impregnated with gin, both to the senses of smell and taste, and even to the test of inflammability. The liquid appeared to the senses of the examining students as strong as one-third gin to two-thirds water." In the 40th vol. of the Edinburgh Medical and Surgical Journal, other cases are recorded by Dr. Ogston, of Aberdeen, in his valuable paper "On the phenomena of the more advanced stages of Intoxication." We find, also, a case, bearing on the same point, in the 1st. vol. of the London Lancet for 1836–37, p. 271, in which, after the exhibition of half an ounce of sulphuric ether, "a strong smell of ether was perceived," on removing the skull cap. "Very moderate effusion in the lateral and fourth ventricles, which smelt strongly of ether, and a piece of lint soaked in in it, and held in the flame of a candle, was thought by the gentlemen present to blaze up rather more than a piece of lint dipped in water, the experiment being made with water also at the same time." Lastly, Dr. Copland, speaking on this subject, observes, " Dr. Ogston confirms the testimony of Wepper, Voight, Carlisle, and others, as to the effused fluid being impregnated with alcohol. In describing the appearances in one of his cases, he states, that about four ounces of fluid were found in the ventricles, having all the physical qualities of alcohol, as proved by the united testimony of two other medical men, who saw the body opened, and examined the fluid."* (Dict. Pract. Med., part iii., Art. Drunkenness.)

These statements, at first sight, appear conclusive; but, on a closer examination, it will readily be perceived that they are not of so specific a character as not to admit of being questioned. We, accordingly, find Professor Christison making the following strong objections to them. " In former editions of this work," observes Dr. C., " I have ventured to question the accuracy of such statements, on the one hand, because in animals poisoned with alcohol introduced into the stomach, I never could perceive the smell in any other part of the body; and, on the other hand, because I have several times remarked, in the venous blood and brain of a fresh subject, a smell which a pre-possessed person might have confounded with that of alcohol, although no spirituous liquor had been taken before death. I must still repeat my doubts, chiefly, however, with the view of calling the attention of pathologists to the subject, and of expressing my surprise that those who imagine they have observed so very extraordinary a physiological fact as the transference of a spirituous fluid, not much diluted, from the stomach to the ventricles of the brain, should not have thought it indispensable to substantiate the fact in the only way in which it admits of being substantiated, namely, by chemical analysis. (Work on poisons, p. 853, 3d edition. Edinburgh.)

It was to meet these objections, particularly the last, that Dr. Percy undertook the investigation of the subject; and I think I am justified in stating that he has succeeded in proving, beyond the shadow of a doubt, that alcohol can be detected in the system after poisoning from alcoholic liquors. He found it not only in the brain, but also in the blood,† urine,‡ bile, and liver,—and he connects (perhaps justly) this latter fact with the frequency of diseased liver in habitual drunkards.

Dr. Percy's experiments were chiefly made on dogs, and the symptoms

* It is much to be regretted that Dr. Copland, after speaking so decidedly on the subject, has not informed us how the fluid was *proved* to be alcohol. Dr. Ogston, in his paper already alluded to, is altogether silent on this point, only stating that the effused fluid had " all the physical qualities of alcohol," but what these *physical qualities* were, or how ascertained, he does *not* mention.

† The credit of first detecting alcohol in the blood is, I believe, due to Majendie, (Dict. de Médecine, tome xii., p. 489.) Dr. Trotter, in his book on Drunkenness, urges the probability that "much of the liquor (alcoholic) enters the circulation, and gives there an additional stimulus." In a review of this work, in the Edinburgh Medical and Surgical Journal, the reviewer maintained, " that neither the blood, nor any of the excretions, when examined, give any indication of the presence of alcohol or intoxicating substance." (Vol. I., p. 57.)

‡ Müller, in his work on Physiology, enumerates alcohol as one of the substances which, when taken into the stomach, cannot be detected afterwards in the urine, (Baly's Translation, p. 589.) Berzelius, if I mistake not, has some where made a similar statement. Dr. Percy's experiment on this point was made in the presence of *Professor Traill.*

and post-mortem appearances are recorded with the greatest minuteness. He has, however, given *one* case in which he detected alcohol in the human brain, and we are happy to find that, in this case, "Dr. Christison confirmed the analysis." We may, therefore, expect, in the next edition of the Professor's work, a recantation of his previous opinion, and due credit given to Dr. Percy for "substantiating the fact in the only way in which it admits of being substantiated, namely, by chemical analysis." In none of these experiments was Dr. Percy able to obtain alcohol from the *fluid of the ventricles*, although a sufficient quantity was readily detected in the substance of the brain,—a result in direct variance from the statements of Drs. Cook, Ogston, &c. I conceive, however, that the experiments related under this head are not conclusive, as in no case was there a sufficient quantity of serum obtained to give the analysis a fair trial. This part of the subject, therefore, is still open for investigation. Should Dr. Percy's statement be confirmed by future experiments, it will present a very curious physiological fact, and one not easily explained. Can there be any affinity between *alcohol* and the *cerebral substance?* "We know (says a writer in the British and Foreign Medical Review) that medicinal substances have been detected in the blood, and that the action of many is more directed upon particular organs than others. How far this fact may be explained, on the supposition of a particular affinity of the medicine for the organ, is a subject well worthy of inquiry." This is a wide field for experimental research, and, if properly cultivated, cannot fail to give us many very important results.

There is, perhaps, no subject in physiology that has given rise to more controversy than the *modus operandi* of narcotic poisons; and they are few which are enveloped in greater obscurity. At present, no less than *three* theories exist on the subject.

First, That the effects of these poisons on the nervous system are caused solely by an impression upon the extremities of the nerves of the stomach, or other organ to which they may be applied.

Second, That the effects are produced by the poison entering the circulation, and making its impression upon the *extremities* of the nerves which are distributed to the coats of the blood-vessels.

Third, That all narcotic poisons (not even excepting prussic acid, whose action is almost instantaneous,) exert their deadly influence directly upon the central organ of the nervous system.

This is not the proper place to enter on the various arguments which have been brought forward in support of each of these doctrines. The discrepancy that exists among authors is merely mentioned to show the necessity for a further investigation of the subject. A patient and judicious perseverance in the path of experimental research may, ultimately, lead to a successful solution of some, at least, of the many difficulties with which it is at present beset; and the bringing to light of a single, and, perhaps, simple fact may at length disperse the darkness which surrounds this most interesting physiological question.

I think it may easily be shown that the *direct* modes in which alcohol produces its *narcotic* effects cannot be explained by an appeal to any *one* of the theories which I have mentioned. Such an enquiry, however, would extend this communication to too great a length, and would, moreover, necessarily lead us into a controversial discussion not adapted to the pages of this journal.

